# Serum 5-Methyltetrahydrofolate Status Is Associated with One-Carbon Metabolism-Related Metabolite Concentrations and Enzyme Activity Indicators in Young Women

**DOI:** 10.3390/ijms241310993

**Published:** 2023-07-01

**Authors:** Yoshinori Kubo, Kumiko Shoji, Akiko Tajima, Sayaka Horiguchi, Hideoki Fukuoka, Masazumi Nishikawa, Yasuo Kagawa, Terue Kawabata

**Affiliations:** 1 Faculty of Nutrition, Kagawa Nutrition University, 3-9-21 Chiyoda, Sakado 350-0288, Japan; shoji.kumiko@eiyo.ac.jp (K.S.); tab201@eiyo.ac.jp (A.T.); shori@saitama-med.ac.jp (S.H.); kagawa@eiyo.ac.jp (Y.K.); kawabata@eiyo.ac.jp (T.K.); 2 Division of Anatomy and Cell Biology, Department of Anatomy, Shiga University of Medical Science, Seta Tsukinowa-cho, Otsu 520-2192, Japan; 3 Department of Perinatal Mesenchymal Stem Cell Research, Fukushima Medical University School of Medicine, 1 Hikarigaoka, Fukushima 960-1295, Japan; fukuokah@fmu.ac.jp; 4 Department of Food Management, School of Food, Agricultural and Environmental Sciences, Miyagi University, 2-2-1 Hatadate, Taihaku-ku, Sendai 982-0215, Japan; nishikaw@myu.ac.jp

**Keywords:** one-carbon metabolism, 5-methyltetrahydrofolate, betaine, homocysteine, *S*-adenosylmethionine, *S*-adenosylhomocysteine, transsulfuration pathway, cysteine, choline, methionine

## Abstract

Maintaining optimal one-carbon metabolism (OCM) is essential for health and pregnancy. In this cross-sectional study, folate status was assessed based on 5-methyltetrahydrofolate (5-MTHF) levels, and the association between 5-MTHF and OCM-related metabolites was investigated in 227 female Japanese university students aged 18–25 years. The participants were divided into high and low 5-MTHF groups based on their folate status. Serum samples of the participants were collected while they were fasting, and 18 OCM-related metabolites were measured using stable-isotope dilution liquid chromatography–electrospray tandem mass spectrometry. The association between serum 5-MTHF and OCM-related metabolite concentrations was assessed using Spearman’s rank correlation coefficient. Serum 5-MTHF concentrations were negatively correlated with total homocysteine (tHcy) concentrations and positively correlated with *S*-adenosylmethionine (SAM) and total cysteine (tCys) concentrations. Serum 5-MTHF concentrations demonstrated a stronger negative correlation with tHcy/tCys than with tHcy alone. The negative correlation between betaine and tHcy concentrations was stronger in the low 5-MTHF group than in the high 5-MTHF group. The 5-MTHF status could be linked to Hcy flux into the transsulfuration pathway via SAM. Therefore, the tHcy/tCys ratio may be a more sensitive indicator of the 5-MTHF status than tHcy alone. Furthermore, a low 5-MTHF status can enhance Hcy metabolism via betaine.

## 1. Introduction

**Figure 1:**
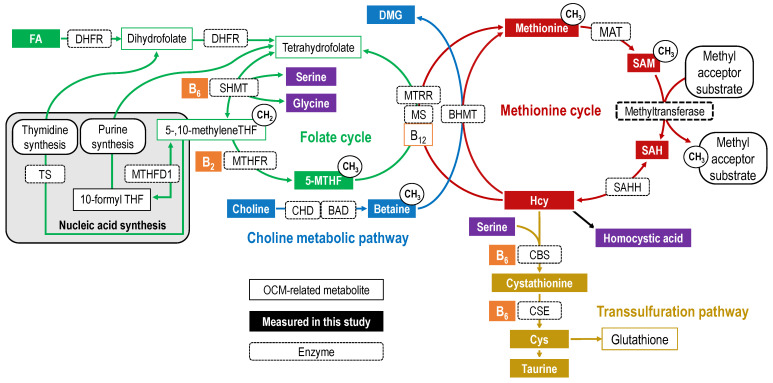
Overview of one-carbon metabolism. OCM-related metabolites are indicated by rectangular boxes, with the folate cycle in green, choline metabolic pathway in blue, methionine cycle in red, and transsulfuration pathway in yellow. Other vitamins are shown in orange and amino acids and others are shown in purple. The filled rectangular boxes indicate OCM-related metabolites measured in this study. Each arrow represents a biochemical reaction, and the dotted rectangle on the arrow is labeled with the first letter of the enzyme catalyzing the reaction. Abbreviations: 5-MTHF, 5-methyltetrahydrofolate; B_12_, cobalamin/methylcobalamin; B_2_, riboflavin; B_6_, pyridoxal phosphate (pyridoxine/pyridoxal/pyridoxamine); BAD, betaine aldehyde dehydrogenase; BHMT, betaine–homocysteine methyltransferase; CBS, cystationine-β synthase; CHD, choline dehydrogenase; CSE, cystathionine γ-lyase; DMG, dimethylglycine; FA, folic acid; MAT, methionine adenosyltransferase; MS, methionine synthase; MTHFD1, methylenetetrahydrofolate dehydrogenase; MTHFR, methylenetetrahydrofolate reductase; MTRR, methionine synthase reductase; SAH, *S*-adenosylhomocysteine; SAHH, SAH hydrolase; SAM, *S*-adenosylmethionine; SHMT, serine hydroxymethyltransferase; THF, tetrahydrofolate; TS, thymidylate synthase.

One-carbon metabolism (OCM) comprises a folate cycle and choline metabolic pathway linked to a methionine cycle; homocysteine (Hcy) in the methionine cycle is connected to the transsulfuration pathway (Figure 1). OCM is mainly involved in the transfer of one-carbon units required for *S*-adenosylmethionine (SAM)-dependent methyl transfer reactions [1], nucleic acid synthesis [1,2], and amino acid metabolism, all of which support numerous physiological processes [2,3].

Folate mediates the transfer of one-carbon units in OCM [2]. Folic acid (FA) in supplements and fortified foods is reduced to tetrahydrofolate (THF) before entering the folate cycle [4]. THF is primarily metabolized to 5,10-methyleneTHF by obtaining the hydroxymethyl group from serine [5]. Subsequently, 5,10-methyleneTHF is irreversibly reduced to 5-methyltetrahydrofolate (5-MTHF) by vitamin B_2_-dependent MTHF reductase (MTHFR; EC 1.5.1.20) [6]. The major folate molecular species is 5-MTHF, accounting for 82 to 93% of the total folate in the blood [7,8]. Methionine synthase (EC 2.1.1.13) uses the methyl group of 5-MTHF to remethylate Hcy to methionine, and demethylated THF is recycled to the folate cycle [3,7,8]. Choline is oxidized to betaine primarily in the liver and kidneys [9]. Betaine is catalyzed by betaine–homocysteine *S*-methyltransferase (BHMT; EC 2.1.1.5), which is expressed primarily in the liver and kidneys and remethylates Hcy to produce methionine and dimethylglycine (DMG) [10,11]. Methionine forms SAM in a reaction catalyzed by methionine adenosyltransferase (EC 2.5.1.6) [12]. SAM is used in methylation reactions that regulate the biological processes of various cellular components and is subsequently metabolized to *S*-adenosylhomocysteine (SAH) [13]. Methylation reactions include epigenetic modifications of gene expression (e.g., methylation of DNA and histones), biosynthesis of molecules (e.g., protein, phosphatidylcholine, polyamines, creatine, and sarcosine), and oxidation–reduction reactions [14,15]. SAH is reversibly degraded to Hcy and adenosine [16]. Both the folate cycle and choline metabolic pathway can independently supply methyl groups for the remethylation of Hcy to methionine; however, these two pathways are considered to be interrelated [17]. Hcy is also irreversibly metabolized to the sulfur-containing amino acid cysteine (Cys) via the transsulfuration pathway in the liver, pancreas, intestine, kidney, and possibly brain [18,19]. Hcy is first metabolized to cystathionine by the B_6_-dependent enzyme cystathionine-β-synthase (CBS; EC 4.2.1.22), which condenses serine to Hcy thiol [18,20]. Then, cystathionine is hydrolyzed by the B_6_-dependent enzyme cystathionine γ-lyase (CSE; EC 4.4.1.1) to Cys [18,21]. Cys is a precursor to sulfur metabolites, such as taurine, glutathione, and hydrogen sulfide [18,19].

OCM metabolic markers have been linked to various diseases. Intracellular Hcy concentrations increase when OCM fails to function adequately [16,22]. The excess Hcy is then excreted from the cells, leading to its increased concentration in the circulation [16,22]. High plasma Hcy concentrations have been linked to cardiovascular diseases [23,24], stroke [25,26], Alzheimer’s disease [27,28], schizophrenia [29,30], macular degeneration [31], diabetes [32], fractures [33], pregnancy complications [34,35,36], small-for-gestational-age offspring [37], and cancer [38]. This suggests that Hcy is an important indicator of health status [39,40]. However, the effects of Hcy-lowering interventions have been modest [41], and whether Hcy is a marker or a causative agent of these diseases remains unclear [39]. Some studies have reported that high plasma SAH concentrations are a considerably more sensitive indicator of cardiovascular diseases than total Hcy (tHcy) concentrations [40]. Homocysteic acid, produced by Hcy and methionine superoxide oxidation, is hypothesized to be an early diagnostic marker for mild cognitive impairment [42]. Low folate status in the blood during early pregnancy increases the risk of fetal neural tube closure defects [43]. In addition, midpregnancy choline status [44] and prepregnancy choline and betaine intake [45] have been linked to the risk of fetal neural tube closure defects. Increased flux in the transsulfuration pathway has been proposed to delay aging and extend life span [46]. During pregnancy, the mother’s OCM status is also important for fetal development and health [47,48]. Therefore, maintaining optimal OCM is essential for health and pregnancy, and thus, a comprehensive understanding of OCM is required.

Previous studies have assessed folate concentrations in circulation using a microbiological assay [49]; however, this assay measures all folate molecular species, including FA and 5-MTHF, and can only assess “total folate” [50,51]. In particular, FA in the blood is biologically inactive and does not accurately reflect its true physiological state, leading to inaccurate and misleading results [52]. Therefore, using liquid chromatography–tandem mass spectrometry is encouraged to quantify individual folate molecular species [49]. Although the total folate concentration in the blood is considered to influence OCM dynamics [53], the association between OCM and 5-MTHF, a major reduced folate, remains unclear. Our study focused on the association between 5-MTHF status and choline metabolism, methionine cycle, and the transsulfuration pathway. A few studies have investigated the metabolic dynamics of OCM [46,54,55,56,57,58,59,60]. However, to the best of our knowledge, no studies have comprehensively investigated the folate cycle, including 5-MTHF, methionine cycle, including SAM and SAH, choline metabolic pathway, or transsulfuration pathway. Furthermore, sex differences in OCM have been reported [54,61,62] because sex hormones, such as estrogen, can upregulate or downregulate various enzymes in OCM [62]. To account for the effects of sex hormones, OCM should be assessed in relation to sex, and women’s characteristics should be further divided into those related to menstruation, pregnancy, and menopause. Therefore, this study aimed to comprehensively measure key OCM-related metabolites in serum collected from young menstruating women and collect basic data regarding the association between 5-MTHF status and OCM.

## 2. Results

### 2.1. Distribution of Serum 5-MTHF Concentrations in Low and High 5-MTHF Groups

**Figure 2:**
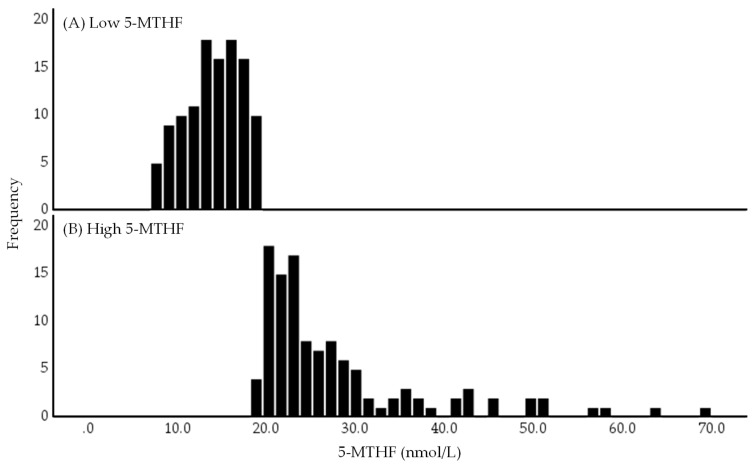
Histograms of low (**A**) and high (**B**) 5-methyltetrahydrofolate (5-MTHF) groups. The low and high 5-MTHF groups were divided based on the median serum 5-MTHF concentration of 19.2 nmol/L. Abbreviations: 5-MTHF, 5-methyltetrahydrofolate.

Figure 2 depicts the histograms of serum 5-MTHF concentrations in the low and high 5-MTHF groups. Serum 5-MTHF concentrations exhibited a positively skewed distribution, with the low 5-MTHF group showing a clumped distribution and the high 5-MTHF group showing a scattered distribution.

### 2.2. Characteristics of the Study Population

The characteristics of the study population are summarized in Table 1. No significant differences in age, height, weight, body mass index (BMI), body fat percentage, or blood pressure were observed between the low and high 5-MTHF groups.

### 2.3. Energy and Nutrient Intakes

Table 2 summarizes the dietary survey results. The high 5-MTHF group had significantly higher energy intake and higher total fiber, potassium, calcium, magnesium, iron, vitamin A, thiamin, vitamin B_6_, folate, and vitamin C intakes, but significantly lower sodium intake than the low 5-MTHF group.

### 2.4. Distribution of Serum OCM-Related Metabolite Concentrations

The serum OCM-related metabolite concentrations in the high and low 5-MTHF groups and in the overall study sample are summarized in Table 3. The high 5-MTHF group exhibited significantly higher betaine and total cysteine (tCys) concentrations and betaine/DMG ratios than the low 5-MTHF group. A similar trend was observed for SAM (*p* = 0.059). However, tHcy and cystathionine concentrations and tCys/tHcy ratios were significantly lower in the high 5-MTHF group than in the low 5-MTHF group. Serum homocysteic acid, pyridoxamine, and pyridoxine concentrations were below the limit of quantification in all samples.

### 2.5. Correlation between Serum OCM-Related Metabolite Concentrations

**Figure 3:**
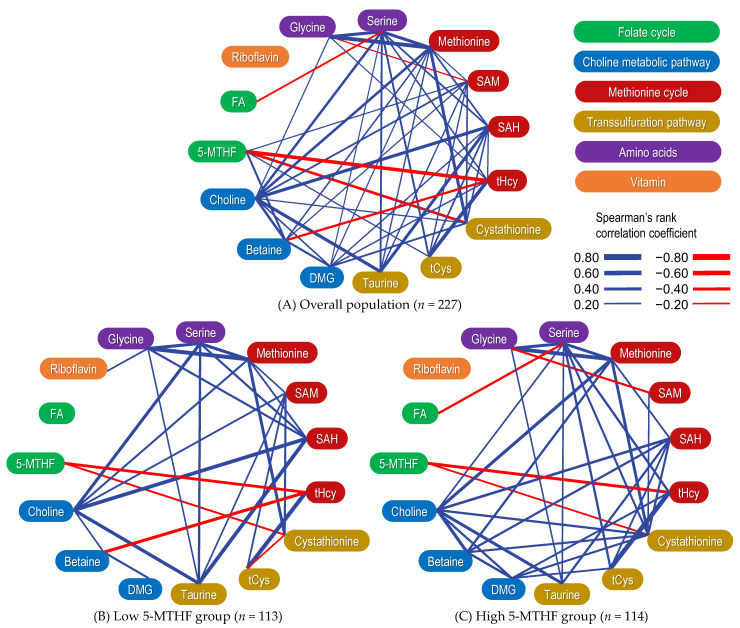
A graphical network depicting the correlation between one-carbon metabolism-related metabolites. Overall population (**A**) as well as low (**B**) and high (**C**) 5-methyltetrahydrofolate (5-MTHF) groups. Correlations were evaluated using Spearman’s correlation coefficient. Significant positive correlations are indicated by blue lines, whereas significant negative correlations are indicated by red lines. The strength of the correlation is indicated by the thickness of the edges. Abbreviations: 5-MTHF, 5-methyltetrahydrofolate; DMG, dimethylglycine; FA, folic acid; SAH, *S*-adenosylhomocysteine; SAM, *S*-adenosylmethionine; tCys, total cysteine; tHcy, total homocysteine.

Supplementary Figure S1 shows correlation matrices, scatter plots, and histograms of serum OCM-related metabolite concentrations. In addition, Figure 3 shows a graphical network depicting their associations. Serum 5-MTHF concentrations exhibited significantly positive correlations with betaine, SAM, and tCys concentrations and significantly negative correlations with tHcy and cystathionine concentrations. Conversely, FA concentrations did not correlate with the serum concentrations of OCM-related metabolites associated with 5-MTHF.

### 2.6. Correlation between OCM-Related Metabolites Stratified by 5-MTHF Status

The correlation matrix between serum OCM-related metabolite concentrations when stratified via dichotomous 5-MTHF concentrations is summarized in Supplementary Table S1, and a graphical network depicting their associations is shown in Figure 3. The negative correlation between betaine and tHcy concentrations was stronger in the low 5-MTHF group (ρ = −0.401, *p* < 0.001) than in the high 5-MTHF group (ρ = −0.097, *p* = 0.303). The positive correlation between DMG and tHcy concentrations was weaker in the low 5-MTHF group (ρ = 0.058, *p* = 0.541) than in the high 5-MTHF group (ρ = 0.249, *p* = 0.007). The correlation matrix between serum OCM-related metabolite concentrations and enzyme activity indices is summarized in Table 4. Similarly, the negative correlation between betaine/DMG ratios and tHcy concentrations was stronger in the low 5-MTHF group (ρ = −0.374, *p* < 0.001) than in the high 5-MTHF group (ρ = −0.290, *p* = 0.002). The 5-MTHF concentrations and betaine/DMG ratios showed a significant positive correlation. The significant negative correlation between 5-MTHF concentrations and tHcy/tCys ratios (ρ = −0.602, *p* < 0.001) was stronger than the correlation between 5-MTHF and tHcy concentrations alone (ρ = −0.456, *p* < 0.001; Supplementary Figure S1).

## 3. Discussion

We measured the serum concentrations of OCM-related metabolites in healthy young women to investigate the association between serum 5-MTHF concentrations and a wide range of OCM-related metabolites. The findings of this study further contribute to preliminary research by demonstrating for the first time that the 5-MTHF state is associated with transsulfuration pathway flux and that betaine-induced Hcy remethylation is enhanced in the low 5-MTHF state.

As expected, the dietary survey results revealed that the high 5-MTHF group exhibited significantly higher folate intake (dietary folate equivalents) than the low 5-MTHF group. On the other hand, the high 5-MTHF group exhibited higher energy and micronutrient intake than the low 5-MTHF group, suggesting a potential dietary influence on OCM. Because food nutrients are complex, it is likely that the intake of folate-rich foods, such as vegetables, altered the intake of other micronutrients. The intake of methionine, cystine, glycine, serine, zinc, riboflavin, and vitamin B_12_, which are OCM components, did not differ significantly between the high and low 5-MTHF groups. However, the high 5-MTHF group exhibited a significantly higher vitamin B_6_ intake, an OCM component, than the low 5-MTHF group; this should be considered when interpreting the study results.

Herein, the median serum 5-MTHF concentration in young Japanese women was 19.2 nmol/L, which was comparable to or slightly higher than that reported in previous studies involving healthy adults in countries where FA fortification of grains is not mandated, such as Japan [63,64]. However, it was lower than that reported in young participants in the 2011–2016 National Health and Nutrition Examination Survey in the United States where grains are fortified with FA [65]. The median serum betaine concentration in this study population was 38.7 µmol/L, consistent with other studies on healthy adults [66,67,68]. Serum pyridoxine, pyridoxamine, and homocysteic acid concentrations were at the limit of quantification in this study. However, similar results have been reported in previous studies, implying that their quantification in serum is difficult [69,70].

Concentrations of 5-MTHF exhibited a significant positive correlation with SAM and tCys concentrations and a significant negative correlation with tHcy concentrations. Similarly, a previous study involving older adults found a positive correlation between 5-MTHF and SAM concentrations, and FA intervention significantly increased plasma SAM concentrations in the intervention group compared with the control group [55]. In addition, previous in-silico studies using mathematical models reported that SAM concentrations demonstrated a strong linear association with total folate concentrations [71]. In-silico studies have also revealed that 5-MTHF is an allosteric inhibitor of glycine N-methyltransferase (EC 2.1.1.20) [72,73], one of the enzymes mediating the methyl transfer reaction. Thus, it is assumed that an increase in 5-MTHF levels increases SAM by inhibiting glycine N-methyltransferase and suppressing the methylation reaction. The low positive correlation coefficient between serum 5-MTHF and SAM concentrations in this study could be attributed to the ability of SAM to maintain its intracellular concentrations by inhibiting MTHFR [74,75], BHMT [76], and methionine adenosyltransferase [77] and activating CBS [78]. In addition, in-silico studies have suggested that the plasma concentrations of OCM-related metabolites do not accurately reflect their intracellular concentrations [79,80]. The negative correlation between blood 5-MTHF and tHcy concentrations has been reported in pregnant women [81], older people [55], and patients with hypertension [82]. Furthermore, the nonlinear negative relationship in patients with hypertension in whom Hcy concentrations are less likely to be low and approach a plateau as serum 5-MTHF concentrations increase [82] is consistent with the findings of this study (Supplementary Figure S1). Interestingly, the mathematical model of the in-silico study estimated that increased remethylation flux does not reduce Hcy concentrations as it circumnavigates the methionine cycle back to Hcy [62]. Furthermore, the authors of the in-silico study concluded that the increased flux in the transsulfuration pathway is due to CBS activation via SAM and betaine [62]. The present study found that the concentrations of tCys, a transsulfuration pathway metabolite, were positively correlated with the concentrations of 5-MTHF, consistent with the results of the in-silico study [62]. In addition, previous studies have reported a positive correlation between serum total folate and plasma tCys concentrations in healthy adults [83,84]. Previous studies have reported that SAM is an allosteric activator of CBS and regulates the flux of Hcy into the transsulfuration pathway [78,85]. Previous studies have also reported that SAH activates CBS [86]. Herein, SAM and SAH concentrations exhibited significant positive correlations with cystathionine, tCys, and taurine concentrations (Figure 2), implying that SAM and SAH may modulate the flux of the transsulfuration pathway in a concentration-dependent manner. However, the correlation coefficients between SAM or SAH and the transsulfuration pathway metabolites (cystathionine, cysteine, and taurine) varied depending on the 5-MTHF state and require further investigation (Supplementary Table S1). Accordingly, the 5-MTHF state may be associated with Hcy flux into the transsulfuration pathway via SAM.

The negative correlation between serum 5-MTHF concentrations and tHcy/tCys ratios was stronger than that between 5-MTHF and tHcy concentrations alone (Supplementary Figure S1 and Table 4). The tHcy/tCys ratio, a substrate/product ratio, is an indicator of enzyme activity (CBS and CSE) in the transsulfuration pathway. Therefore, higher 5-MTHF concentrations may indicate a lower Hcy and higher Cys association. A previous cross-sectional study involving patients aged 21–88 (median, 62) years who had undergone coronary angiography for suspected coronary artery disease or aortic stenosis revealed a stronger negative association between the plasma total folate concentrations and tCys/tHcy ratios than that between plasma total folate concentrations and plasma tHcy concentrations [59]; these findings were consistent with those of the present study. A case–control study reported comparable results in patients with colorectal cancer and matched controls [87]. Therefore, the tHcy/tCys ratio is a more sensitive indicator of the 5-MTHF status than the tHcy concentration. Further investigation into the clinical implications of increased activation of transsulfuration pathways is warranted. Interestingly, in a previous study, increased Hcy concentrations and decreased Cys concentrations were observed in patients with schizophrenia [88], implying that the transsulfuration pathway plays an important role in the etiology of schizophrenia [88]. This theory suggests that schizophrenia may be characterized by impaired glutathione synthesis and increased susceptibility to oxidative stress. In other words, the pathological hypothesis of schizophrenia is centered on increased oxidative stress and impaired antioxidant function [89]. A previous study also reported that high plasma Hcy concentrations were associated with increased colorectal cancer risk, whereas high Cys concentrations were associated with a lower colorectal cancer risk [89] and that Hcy/Cys ratios were inversely correlated with colorectal cancer risk [87]. Conversely, studies have suggested that high plasma tCys concentrations in healthy women may cause more damage to the vascular endothelium than tHcy [90]. A similar association between betaine and tHcy/tCys ratio has also been shown (Appendix A.1). Therefore, the optimal state of the transsulfuration pathway, including 5-MTHF and betaine, should be investigated to clarify these findings.

Herein, 5-MTHF concentrations exhibited significant positive correlations with betaine concentrations or betaine/DMG ratios (Figure 3 and Table 4). Similarly, previous studies have reported a positive correlation between blood total folate and betaine concentrations [68,91]. According to in-silico studies, SAM concentrations increase with increasing total folate concentrations, SAM inhibits BHMT, and betaine is less likely to be metabolized, resulting in increased betaine concentrations [71]. In addition, previous intervention studies have reported that FA supplementation increases plasma betaine concentrations in both healthy adults [66] and older individuals [92]. Therefore, the findings of this study support those of previous studies that reported an association between total folate concentrations and betaine metabolism. Furthermore, when the cohort was divided into diquantiles based on serum 5-MTHF concentrations, the negative correlation between betaine concentrations or betaine/DMG ratios and tHcy concentrations was stronger in the low 5-MTHF group than in the high 5-MTHF group (Figure 3 and Table 4). Conversely, the positive correlation between tHcy and DMG concentrations was weaker in the low 5-MTHF group than in the high 5-MTHF group. Previous studies have reported that blood betaine concentrations inversely correlate with Hcy concentrations in healthy adults [66,68,93], clinic attendees [94], pregnant women [95], patients with cardiovascular disease [96,97], and patients with chronic renal failure [98], indicating that betaine and folate are important determinants of plasma Hcy concentrations. If the 5-MTHF concentration is low, the supply of methyl groups from 5-MTHF to Hcy may be insufficient for Hcy to remethylate methionine, thus necessitating another Hcy methyl group donation pathway, i.e., betaine to Hcy. In a previous study, when pregnant women were divided into diquantiles based on plasma total folate concentration, the low folate group exhibited higher plasma DMG concentrations and lower plasma betaine concentrations than the high folate group [99]. An interventional study involving healthy men and women reported that an increase in plasma Hcy concentrations following methionine loading was inversely correlated with plasma betaine concentrations and that this association was stronger in patients with low blood folate concentrations than in those with high blood folate concentrations [68,93]. The findings of previous studies evaluating total folate states are consistent with those of the present study [68,93]. As a mechanism, methionine synthase expression has been reported to decrease in the liver of mice fed with a low folate diet [100], and decreased SAM concentrations have been reported to activate BHMT in the rat liver [101]. Certainly, the present study showed a weak positive correlation between SAM and betaine (Appendix A.2). A previous study also estimated a decrease in SAM concentrations and BHMT response activation in the liver using a mathematical model based on known enzyme kinetics, assuming a 50% decrease in folate concentrations [53]. As 5-MTHF can alter the association of tHcy with betaine or DMG, the betaine/DMG ratio, a measure of BHMT activity, may serve as a good indicator of Hcy remethylation via the choline metabolic pathway. Conversely, in-silico studies have reported that the negative association between betaine and tHcy concentrations in women is due to CBS activation based on betaine concentrations rather than a faster BHMT reaction [62]. In other words, CBS activation as a result of high betaine concentrations is less likely to occur when 5-MTHF concentrations are high. Thus, the 5-MTHF status may affect the choline metabolic pathway (Appendix A.3), particularly BHMT or CBS activity, via betaine concentrations in low 5-MTHF conditions, implying that the folate cycle and choline metabolic pathway should be evaluated simultaneously in Hcy studies.

Herein, a negative association between serum 5-MTHF and cystathionine concentrations was observed (Figure 3). A negative correlation between blood total folate and cystathionine concentrations has also been observed in neonates [102] and patients with coronary artery disease [103,104], possibly because Hcy and cystathionine have similar kinetics [104]. This correlation was different than that observed between tHcy and tCys, and the complex transsulfuration pathway needs to be further elucidated.

Serum FA concentrations were not correlated with OCM-related metabolite concentrations associated with 5-MTHF concentrations. This suggests that the concentration of 5-MTHF, a bioactive folate molecule species, as a biomarker may be more useful than that of total folate, which was considered in previous studies [49].

This study has several strengths. Early morning fasting blood samples were collected, and standardized blood collection protocols were used to minimize the following effects on OCM-related metabolites: changes in the blood concentration of the measured substance due to eating immediately before the blood sample is collected; errors related to human activities at the time of blood collection; and variation in the deterioration of the substance being measured. Furthermore, the serum concentrations of OCM-related metabolites were measured using the stable-isotope dilution mass spectrometry method with internal standards for all measured components, yielding high-quality quantitative results [105].

However, this study has several limitations. First, given the cross-sectional survey design, it is impossible to establish a causal association between OCM metabolic dynamics. Further intervention studies on FA or 5-MTHF are required. Second, serum OCM-related metabolites do not always directly reflect OCM dynamics in organ cells. Third, 5-MTHF-dependent Hcy remethylation is a vitamin B_12_-dependent reaction, and vitamin B_12_ is an important determinant of Hcy [106,107]; however, it was not measured in this study. Fourth, recruitment and regional influences, such as only including women from one university who majored in nutrition, might have introduced a sampling bias. Furthermore, FA-fortified rice was routinely served in the university cafeteria. Therefore, generalizing the findings to the Japanese population should be considered with caution. Fifth, Hcy is influenced by numerous factors, including lifestyle [106]; however, confounding factors influencing blood Hcy concentrations were left unadjusted. Sixth, while postprandial OCM kinetic variability was minimized by fasting, the potential impact of habitual diet cannot be ruled out [108,109]. In particular, vitamin B_6_ intake may have promoted transsulfuration pathway flux. Finally, single-nucleotide polymorphisms may have an impact on OCM [56]; however, they have not been considered.

## 4. Materials and Methods

### 4.1. Study Design

This study was conducted at Kagawa Nutrition University, Saitama, Japan, between October and December 2018. This was a cross-sectional study designed to investigate the association between diet and blood components such as biochemical test values, OCM-related metabolites, fatty acids, and antioxidants; we reported associations between serum OCM-related metabolites.

### 4.2. Participants

Healthy female university students aged 18–25 years majoring in nutrition were included. The following students were excluded: (1) those with health conditions that may affect the biomarker concentration; (2) those with a history of, or who have serious hepatic, renal, cardiac, pulmonary, gastrointestinal (including gastrectomy), or organ disorders; diabetes; food allergies; or other serious diseases; (3) those receiving medicines that can affect lipid metabolism or FA metabolism or anti-inflammatory and antioxidant drugs that may affect the measured values in this study; (4) those pregnant and lactating or planning for pregnancy and lactation; (5) those with systolic blood pressure <90 mmHg; (6) those who had previously donated large amounts of blood; (7) those participating in other clinical trials or studies or within 4 weeks of the end of those studies; (8) those with BMI >25 because BMI may affect blood SAM and SAH levels [110,111]; and (9) those judged by the principal investigator to be ineligible for this study. The participants were recruited via a university bulletin board.

A total of 258 eligible participants consented to the study, including proxy consent for minors. After the study began, 31 participants dropped out owing to consent withdrawals (*n* = 9), lost contact (*n* = 6), health problems (*n* = 1), busyness (*n* = 4), and withdrawals (*n* = 11), leaving 227 participants. The participants completed a lifestyle questionnaire and underwent physical measurements, and their fasting blood samples were collected after at least 10 h of fasting.

### 4.3. Ethics

The study protocol was approved by the Ethics Committee of Kagawa Nutrition University (protocol code no. 204) and was conducted in accordance with the Helsinki Declaration. All participants provided written informed consent before participating in the study.

### 4.4. Sample Collection and Processing

To minimize dietary effects [63,67,112], the study participants were instructed to stop eating and drinking by 10 pm and consume nothing but water until blood collection. The participants visited the laboratory in the morning, where 15 mL of venous blood was collected in the supine position and delivered into a serum separation tube. The blood was allowed to clot at room temperature for 30 min, centrifuged at 4 °C for 10 min at 2000× *g*, and the serum was then separated within an hour and frozen at −80 °C. Under these storage conditions, the concentrations of OCM-related metabolites measured in this study have been reported to be generally stable [51,113,114].

### 4.5. Measurement of Serum OCM-Related Metabolites

We previously described the method for measuring OCM-related metabolites [81]. After combining the methods of Guiraud et al. [70] and Zheng et al. [115], we set up multiple reaction monitoring transitions for 18 OCM-related metabolites and their corresponding 18 internal standards (*m*/*z* 460.2→313.2 (5-MTHF), *m*/*z* 465.2→313.2 (5-MTHF^−13^C_5_), *m*/*z* 442.2→295.2 (FA), *m*/*z* 447.2→295.2 (FA^−13^C_5_), *m*/*z* 104.0→60.1 (choline), *m*/*z* 113.0→69.1 (choline-d_9_), *m*/*z* 118.1→58.0 (betaine), *m*/*z* 129.0→66.1 (betaine-d_11_), *m*/*z* 104.1→58.1 (DMG), *m*/*z* 110.1→64.0 (DMG-d_6_), *m*/*z* 150.1→104.0 (methionine), *m*/*z* 153.0→107.0 (methionine-d_3_), *m*/*z* 399.1→250.3 (SAM), *m*/*z* 402.2→250.2 (SAM-d_3_), *m*/*z* 385.2→134 (SAH), *m*/*z* 389.2→136.2 (SAH-d_4_), *m*/*z* 136.0→90.0 (Hcy), *m*/*z* 140.0→93.9 (Hcy-d_4_), *m*/*z* 184.1→138.3 (homocysteic acid), *m*/*z* 188.1→142.0 (homocysteic acid-d_4_), *m*/*z* 223.2→134.1 (cystathionine), *m*/*z* 227.1→138.1 (cystathionine-d_4_), *m*/*z* 122.1→59.1 (Cys), *m*/*z* 124.2→61.0 (Cys-d_2_), *m*/*z* 126.1→107.8 (taurine), *m*/*z* 128.1→110.2 (taurine^−13^C_2_), *m*/*z* 106.1→60.1 (serine), *m*/*z* 109.1→63.1 (serine-d_3_), *m*/*z* 76.0→30.0 (glycine), *m*/*z* 78.0→31.9 (glycine-d_2_), *m*/*z* 377.1→243.1 (riboflavin), *m*/*z* 383.2→249.1 (riboflavin^−13^C_4_^15^N_2_), *m*/*z* 169.2→152.2 (pyridoxamine), *m*/*z* 172.1→155.1 (pyridoxamine-d_3_), *m*/*z* 170.1→134.1 (pyridoxine), and *m*/*z* 172.1→136.0 (pyridoxine-d_2_)) [105]. Hcy and Cys were detected in reduced forms as tHcy and tCys, respectively, under the influence of reducing agents. For liquid chromatography, an Agilent 1200 Series (Agilent Technologies, Tokyo, Japan) was used, and the ion source was a Turbo Ion Spray (Applied Biosystems SCIEX, Tokyo, Japan). The triple quadrupole mass spectrometer was a 4000 QTRAP System (Applied Biosystems SCIEX). The measurement time was 13 min, and the mobile phase flow rate was 500 μL/min, with “A” being a 5 mmol/L perfluoroheptanoic acid solution, “B” being an acetonitrile gradient, and the separation column being an XSelect HSS T3 Column, 2.5 μm, 100 × 2.1 μm (Nihon Waters, Tokyo, Japan). The measurements were taken twice, and the mean value was used. For precision control, eight-point calibration curves were created every 24 h, and quality control measurements were taken every 12 h. The intra-assay and interassay coefficients of variation were 0.3% to 9.1% and 0.5 to 13.5%, respectively. When measuring, control sera were under low-concentration conditions in a preliminary validity test [116]. Analyst 1.6.3 software was employed to process and quantify the data. The concentration value was 0 if the peak could not be detected or the signal-to-noise ratio was <10 as the quantification limit.

### 4.6. Attribute Data

Data regarding the participants’ ages were collected using a self-administered questionnaire.

### 4.7. Nutrient Intake

Dietary data were collected for 7 days before blood collection using a continuous, non-weighted dietary record in conjunction with digital images captured using a digital camera or smartphone according to a validated dietary survey method [117,118,119]. In brief, the participants were instructed to photograph all their meals except water while writing the menu name, ingredients used, and portion size on a self-administered food record form. The photographs were captured before and after the meal, and the participants were asked to place a designated card that would serve as a scale to measure the food. If they ate out or consumed products, they were asked to record the restaurant and brand names of the food. Trained nutrition students and researchers used digital images and dietary records to infer the type and weight of individual food items consumed by the participants based on the standardized Dietary Survey Manual [120] and obtained additional information from the participants when information was unclear. Individual food items were coded based on Japan’s Standard Tables of Food Composition, 2015 [121], and food codes that best approximated the state of the food at the time of eating were selected. Estimated energy and nutrient intakes were calculated using Excel eiyoukun, Version 8.2 (Kenpakusha, Tokyo, Japan). Nutrient intakes were averaged over 7 days, and energy was adjusted for each nutrient intake using the density method.

### 4.8. Blood Pressure Measurement

Blood pressure was measured using a P2000 Electronic Blood Pressure Monitor (TERUMO, Yamanashi, Japan). The mean systolic and diastolic blood pressure was measured twice in the resting state according to the Japanese guidelines for managing hypertension [122].

### 4.9. Anthropometric Data

The participants’ anthropometric measurements were collected using standardized methods during laboratory visits for blood sampling. Body weight and body fat percentage were measured using TBF-110 (TANITA, Nagano, Japan).

### 4.10. Statistical Analysis

The distribution of subject characteristics data, nutrient intake, and serum concentrations of OCM-related metabolites used in the analysis were mostly non-normal and continuous variables were expressed as medians (25–75th percentile values). Since there are no thresholds established for serum 5-MTHF concentrations such as deficiency, insufficiency, sufficiency, or excess, based on a previous study [99], a median serum 5-MTHF concentration of 19.2 nmol/L was used to stratify the participants into high and low 5-MTHF groups, and the Mann–Whitney *U* test was used to compare these groups. The enzyme activity indices included the betaine/DMG ratio [98] for BHMT activity, SAM/SAH ratio [54] for methyltransferase activity, and tCys/tHcy ratio [59] for enzyme activity in the transsulfuration pathway (CBS and CSE). The significance level was set at a two-tailed *p*-value of <0.05. IBM Statistical Package for the Social Sciences Statistics, Version 28 (IBM, Japan) was used for statistical analysis.

## 5. Conclusions

Previous studies assessed folate status without considering folate molecular species. Thus, we extended the findings of previous studies by comprehensively measuring OCM-related metabolites, including 5-MTHF, a major reduced folate. FA showed no association with important OCM-related metabolites, indicating that blood 5-MTHF levels could be useful for evaluating folate status. The 5-MTHF state could be linked to the Hcy flux into the transsulfuration pathway via SAM. Therefore, the tHcy/tCys ratio may be a more sensitive indicator of the 5-MTHF status than tHcy concentrations alone. A low 5-MTHF status may affect the choline metabolic pathway, particularly BHMT or CBS activity via betaine, implying that the folate cycle and choline metabolic pathway should be evaluated simultaneously in Hcy studies. Further intervention studies on FA or 5-MTHF are warranted to determine the causal association between the abovementioned findings.

## Figures and Tables

**Table 1:** Characteristics of the study population.

Variables	Overall Population (*n* = 227)			Low 5-MTHF Group (*n* = 113)			High 5-MTHF Group (*n* = 114)			
	Median	25th	75th	Median	25th	75th	Median	25th	75th	*p*-Value ^a^
Age (years)	20	19	21	20	19	21	20	19	21	0.873
Height (cm)	158	155	162	159	155	162	158	155	163	0.858
Body weight (kg)	51.2	47.4	55.3	50.8	47.0	55.3	51.6	47.5	55.4	0.887
BMI (kg/m^2^)	20.2	19.1	21.6	20.1	19.1	21.8	20.3	19.0	21.4	0.855
Body fat percentage (%)	24.9	22.5	28.4	24.9	23.0	28.4	24.9	22.2	28.3	0.480
Mean systolic blood pressure (mmHg)	106.5	100.0	114.5	106.5	100.5	113.3	106.8	99.0	116.1	0.985
Mean diastolic blood pressure (mmHg)	68.0	64.0	74.0	68.5	65.0	73.5	67.8	62.5	75.1	0.474

^a^ *p*-values were calculated using Mann–Whitney *U* test (low vs. high 5-MTHF groups). There were no missing values. Abbreviations: BMI, body mass index; 5-MTHF, 5-methyltetrahydrofolate.

**Table 2:** Energy and nutrient intakes of the subjects.

	Overall Population (*n* = 227)			Low 5-MTHF Group (*n* = 113)			High 5-MTHF Group (*n* = 114)			
	Median	25th	75th	Median	25th	75th	Median	25th	75th	*p*-Value ^a^
Energy (kcal)a	1731	1460	1964	1672	1408	1896	1797	1496	2082	0.110
Protein (% energy)	14.2	13.1	15.5	14.0	12.9	15.3	14.3	13.3	15.8	0.222
Fat (% energy)	31.9	28.5	35.1	31.6	28.3	35.4	32.0	29.2	34.2	0.799
Carbohydrate (% energy)	52.4	49.0	55.5	51.9	48.8	55.5	52.4	49.3	55.3	0.942
Methionine (mg/1000 kcal)	740	657	832	730	631	823	745	663	840	0.566
Cystine (mg/1000 kcal)	489	452	527	492	453	522	489	446	535	0.841
Total sulfur-containing amino acids (mg/1000 kcal)	1229	1113	1351	1223	1096	1348	1233	1113	1355	0.582
Glycine (mg/1000 kcal)	1448	1286	1625	1452	1297	1623	1442	1276	1628	0.998
Serine (mg/1000 kcal)	1547	1405	1665	1546	1394	1646	1551	1413	1696	0.327
Saturated fatty acids (% energy)	10.0	8.6	11.2	9.8	8.4	11.5	10.1	8.7	11.1	0.997
Polyunsaturated fatty acids (g/1000 kcal)	6.53	5.76	7.27	6.48	5.56	7.22	6.65	5.86	7.42	0.109
n-3 polyunsaturated fatty acids (g/1000 kcal)	1.02	0.85	1.23	0.99	0.83	1.22	1.03	0.85	1.23	0.297
n-6 polyunsaturated fatty acids (g/1000 kcal)	5.44	4.80	6.11	5.43	4.64	6.04	5.47	4.96	6.18	0.099
Total fiber (g/1000 kcal)	7.09	6.16	8.34	6.68	5.81	7.76	7.67	6.79	8.81	<0.001
Sodium (mg/1000 kcal)	1911	1681	2220	2006	1692	2353	1855	1662	2123	0.025
Potassium (mg/1000 kcal)	1092	973	1228	1051	943	1139	1176	1025	1287	<0.001
Calcium (mg/1000 kcal)	258	223	311	252	206	296	266	230	321	0.006
Magnesium (mg/1000 kcal)	118	105	135	113	99	126	126	111	138	<0.001
Iron (mg/1000 kcal)	3.76	3.32	4.16	3.55	3.19	4.05	3.84	3.46	4.27	0.001
Zinc (mg/1000 kcal)	4.12	3.77	4.51	4.11	3.80	4.57	4.15	3.73	4.51	0.907
Vitamin A (μg retinol activity equivalent/1000 kcal)	243	196	295	235	189	278	258	205	326	0.011
Vitamin D (µg/1000 kcal)	2.28	1.37	3.32	2.12	1.46	3.35	2.34	1.33	3.34	0.589
Thiamin (mg/1000 kcal)	0.498	0.433	0.612	0.476	0.419	0.597	0.518	0.448	0.663	0.010
Riboflavin (mg/1000 kcal)	0.596	0.538	0.684	0.573	0.525	0.674	0.613	0.550	0.698	0.094
Vitamin B_6_ (mg/1000 kcal)	0.568	0.494	0.667	0.537	0.460	0.626	0.631	0.535	0.706	<0.001
Vitamin B_12_ (μg/1000 kcal)	2.49	1.62	3.76	2.42	1.63	3.78	2.56	1.54	3.71	0.716
Folate (μg dietary folate equivalents/1000 kcal)	144	123	170	136	113	160	162	135	187	<0.001
Vitamin C (mg/1000 kcal)	41.9	32.4	54.2	37.1	28.2	48.3	44.2	35.5	59.7	<0.001

Values are expressed as medians and 25th–75th percentile values. ^a^ *p*-values were calculated using Mann–Whitney *U* test (low vs. high 5-MTHF groups). Abbreviations: 5-MTHF, 5-methyltetrahydrofolate.

**Table 3:** Concentrations of one-carbon metabolism-related metabolites.

		Overall Population (*n* = 227)			Low 5-MTHF Group (*n* = 113)			High 5-MTHF Group (*n* = 114)			
	Analytes (unit)	Median	25th	75th	Median	25th	75th	Median	25th	75th	*p*-Value ^b^
Folate cycle	5-MTHF (nmol/L)	19.2	14.4	24.2	14.4	11.8	16.7	24.1	21.4	29.6	-
	FA (nmol/L)	1.08	0.62	1.91	0.99	0.59	1.95	1.18	0.74	1.89	0.503
Choline metabolic pathway	Choline (µmol/L)	7.56	6.55	8.43	7.44	6.41	8.30	7.58	6.69	8.61	0.299
	Betaine (µmol/L)	38.7	32.9	45.5	37.7	32.0	42.5	40.9	34.5	47.5	0.004
	DMG (µmol/L)	2.97	2.50	3.60	3.01	2.52	3.78	2.91	2.40	3.48	0.207
	Betaine/DMG	13.2	10.5	15.4	12.4	10.0	14.4	13.9	11.5	16.9	<0.001
Methionine cycle	Methionine (µmol/L)	24.2	22.0	26.9	24.5	22.0	28.0	24.0	22.0	26.6	0.302
	SAM (nmol/L)	55.6	50.7	60.6	54.5	48.8	60.2	56.9	51.6	61.3	0.059
	SAH (nmol/L)	14.5	12.1	17.1	14.8	11.5	17.4	14.4	12.4	17.1	0.928
	SAM/SAH	3.92	3.15	4.96	3.88	3.11	4.95	3.97	3.22	5.04	0.732
	tHcy (µmol/L)	6.39	5.53	7.41	6.92	5.89	7.96	6.07	5.21	6.75	<0.001
	Homocysteic acid (µmol/L)	0 ^a^	0 ^a^	0 ^a^	0 ^a^	0 ^a^	0 ^a^	0 ^a^	0 ^a^	0 ^a^	-
Transsulfuration pathway	Cystathionine (nmol/L)	90.0	73.5	111.0	96.4	80.7	116.5	84.0	68.9	100.6	<0.001
	tCys (µmol/L)	199	185	211	194	181	208	203	191	213	0.009
	tHcy/tCys	0.0322	0.0287	0.0370	0.0354	0.0308	0.0400	0.0302	0.0265	0.0328	<0.001
	Taurine (µmol/L)	112	97	129	111	99	131	113	96	127	0.633
Amino acids	Serine (µmol/L)	146	130	167	144	129	164	151	130	168	0.421
	Glycine (µmol/L)	202	180	225	205	179	229	198	180	221	0.402
Vitamins	Riboflavin (nmol/L)	11.8	7.9	17.5	11.0	8.0	17.0	12.5	7.8	17.6	0.415
	Pyridoxamine (nmol/L)	0 ^a^	0 ^a^	0 ^a^	0 ^a^	0 ^a^	0 ^a^	0 ^a^	0 ^a^	0 ^a^	-
	Pyridoxine (nmol/L)	0 ^a^	0 ^a^	0 ^a^	0 ^a^	0 ^a^	0 ^a^	0 ^a^	0 ^a^	0 ^a^	-

^a^ Below the limit of quantification. ^b^ *p*-values were calculated using Mann–Whitney *U* test (low vs. high 5-MTHF groups). Abbreviations: 5-MTHF, 5-methyltetrahydrofolate; DMG, dimethylglycine; FA, folic acid; SAH, *S*-adenosylhomocysteine; SAM, *S*-adenosylmethionine; tCys, total cysteine; tHcy, total homocysteine.

**Table 4:** Correlation matrices between serum one-carbon metabolite-related metabolite concentrations and enzyme activity indices.

	Enzyme Activity Indices	5-MTHF	FA	Choline	Betaine	DMG	Methionine	SAM	SAH	tHcy	Cystathionine	tCys	Taurine	Serine	Glycine	Riboflavin
Overall population (*n* = 227)	Betaine/DMG	0.284 **	0.073	0.062	0.503 **	−0.642 **	0.020	−0.028	−0.084	−0.385 **	−0.158 *	−0.110	−0.034	−0.032	−0.001	0.127
	SAM/SAH	0.059	0.132 *	−0.299 **	−0.053	−0.095	−0.177 **	0.313 **	−0.895 **	−0.149 *	−0.090	−0.154 *	−0.289 **	−0.237 **	−0.174 **	−0.063
	tHcy/tCys	−0.602 **	−0.007	0.025	−0.324 **	0.120	0.022	−0.090	0.086	0.877 **	0.189 **	0.034	0.128	0.086	0.090	−0.071
Low 5-MTHF group (*n* = 113)	Betaine/DMG	0.173	0.072	0.076	0.498 **	−0.654 **	−0.032	−0.001	−0.212 *	−0.374 **	−0.164	−0.052	−0.057	−0.066	−0.087	0.103
	SAM/SAH	0.091	0.269 **	−0.388 **	0.107	−0.081	−0.174	0.248 **	−0.883 **	−0.147	−0.003	−0.095	−0.389 **	−0.261 **	−0.309 **	−0.149
	tHcy/tCys	−0.450 **	−0.057	−0.022	−0.444 **	0.045	−0.030	−0.064	0.119	0.894 **	0.037	0.136	0.126	0.083	0.086	−0.010
High 5-MTHF group (*n* = 114)	Betaine/DMG	0.150	0.053	0.037	0.461 **	−0.641 **	0.105	−0.108	0.027	−0.290 **	−0.027	−0.239 *	−0.010	−0.026	0.112	0.093
	SAM/SAH	0.065	−0.019	−0.197 *	−0.213 *	−0.110	−0.174	0.398 **	−0.900 **	−0.159	−0.189 *	−0.219 *	−0.188 *	−0.195 *	−0.042	0.001
	tHcy/tCys	−0.439 **	0.059	0.137	−0.087	0.146	−0.014	−0.022	0.074	0.854 **	0.123	0.144	0.131	0.184	0.067	−0.093

Spearman’s correlation coefficient; asterisks indicate the statistical significance of correlation coefficients: ** *p* < 0.01; * *p* < 0.05. The enzyme activity indices were the betaine/DMG ratio for BHMT, SAM/SAH ratio for methyltransferase, and tCys/tHcy ratio for the transsulfuration pathway (CBS and CSE). Abbreviations: 5-MTHF, 5-methyltetrahydrofolate; BHMT, betaine–homocysteine methyltransferase; CBS, cystathionine-β synthase; CSE, cystathionine γ-lyase; DMG, dimethylglycine; FA, folic acid; SAH, *S*-adenosylhomocysteine; SAM, *S*-adenosylmethionine; tCys, total cysteine; tHcy, total homocysteine.

## Data Availability

The dataset used in this study is available upon reasonable request.

## Supplementary Materials

The supporting information can be downloaded at: https://www.mdpi.com/article/10.3390/ijms241310993/s1.

## Abbreviations

5-MTHF	5-methyltetrahydrofolate
BHMT	Betaine–homocysteine S-methyltransferase
BMI	Body mass index
CBS	Cystathionine β-synthase
CSE	Cystathionine γ-lyase
Cys	Cysteine
DMG	Dimethylglycine
FA	Folic acid
Hcy	Homocysteine
MAT	Methionine adenosyltransferase
MTHFR	Methylenetetrahydrofolate reductase
OCM	One-carbon metabolism
SAH	*S*-adenosylhomocysteine
SAM	*S*-adenosylmethionine
tCys	Total cysteine
tHcy	Total homocysteine
THF	Tetrahydrofolate

## Appendix A

### Appendix A.1. Association between Betaine Concentrations and tHcy/tCys Ratio

Similar to the correlation between 5-MTHF concentrations and tHcy/tCys ratios, the correlation between betaine concentrations and tHcy/tCys ratios (ρ = −0.324, Table 3) was stronger than that between betaine and tHcy concentrations alone (ρ = −0.292, Supplementary Table S1). This was also reported in a previous study [59], and the tHcy/tCys ratio could serve as a sensitive indicator of the betaine status and a simple indicator of the complex folate cycle and choline metabolic pathway.

### Appendix A.2. Association between Betaine and SAM Concentrations

Similar to 5-MTHF, betaine concentrations that remethylate Hcy had a significant positive correlation with SAM concentrations. A previous study also reported that betaine concentrations positively correlated with SAM concentrations in healthy men and women [123], indicating the significance of considering betaine status in addition to 5-MTHF status in OCM methyl group metabolism. However, the association between betaine and SAM could be indirect, since 5-MTHF and betaine are positively correlated.

### Appendix A.3. 5-MTHF Status and its Association with Choline Metabolic Pathways

Herein, significant correlations were detected between choline and betaine, choline and DMG, and betaine and DMG concentrations, with a stronger correlation in the high 5-MTHF group than in the low 5-MTHF group (Supplementary Table S1). These associations have also been reported in previous studies involving pregnant women and healthy adults [67,68,93], and they may be explained by the fact that choline is a precursor to betaine and DMG [68]. The findings of the present study imply that the association between these choline metabolic pathways may be influenced by the 5-MTHF status.
