# Supplementary material for: Serum 5-Methyltetrahydrofolate Status Is Associated with One-Carbon Metabolism-Related Metabolite Concentrations and Enzyme Activity Indicators in Young Women
Source: Int J Mol Sci. 2023 Jul 1;24(13):10993. doi: 10.3390/ijms241310993 (PMC10341762; doi:10.3390/ijms241310993)
Supplement: Supplementary file 1 [file ijms-24-10993-s001.zip › SUP-1.pdf]

		5-MTHF	FA	Choline	Betaine	DMG	Methionine	SAM	SAH	tHcy	Cystathionine	tCys	Taurine	Serine	Glycine	Riboflavin
Folate cycle	5-MTHF															
	FA	0.066														
Choline metabolic pathway	Choline	0.108	-0.036													
	Betaine	0.250**	-0.003	0.314**												
	DMG	-0.102	-0.040	0.191**	0.261**											
Methionine cycle	Methionine	-0.075	-0.081	0.316**	0.183**	0.136*										
	SAM	0.138*	0.033	0.221**	0.139*	0.117	0.131*									
	SAH	-0.019	-0.110	0.408**	0.108	0.160*	0.213**	0.077								
	tHcy	-0.456**	-0.046	0.058	-0.292**	0.171**	0.026	-0.019	0.156*							
Transsulfuration pathway	Cystathionine	-0.341**	-0.023	0.151*	0.064	0.232**	0.384**	0.198**	0.186**	0.121						
	tCys	0.149*	-0.050	0.120	0.006	0.087	0.033	0.162*	0.210**	0.460**	-0.078					
	Taurine	-0.025	-0.057	0.408**	0.071	0.077	0.161*	0.162*	0.383**	0.123	0.066	0.094				
Amino acids	Serine	0.026	-0.202**	0.305**	0.098	0.133*	0.392**	-0.037	0.207**	0.183**	0.106	0.240**	0.239**			
	Glycine	-0.079	-0.077	0.135*	0.029	0.052	0.514**	-0.137*	0.112	0.134*	-0.003	0.081	0.125	0.402**		
Vitamin	Riboflavin	0.064	0.089	0.104	0.050	-0.105	-0.028	0.099	0.113	-0.036	-0.106	0.039	0.067	-0.042	0.077	

Figure S1. Correlation matrices between serum OCM-related metabolite concentrations.

The histogram is drawn in the shaded area.

Lower left shows spearman correlation coefficient, asterisks indicate statistical significance of correlation coefficients as follows ** $p < 0.01$; * $p < 0.05$.

The upper right is a scatter plot of the correlation coefficients shown in the lower left.

The sample size was $n = 227$ and there were no missing values.

Serum homocysteic acid, pyridoxamine, and pyridoxine concentrations were below the limit of quantitation in all samples, so results are not shown.

Abbreviations, 5-MTHF; 5-methyltetrahydrofolate; FA; folic acid; DMG; dimethylglycine; SAM; *S* -adenosylmethionine; SAH; *S* -adenosylhomocysteine; tHcy; total homocysteine; tCys; total cysteine.
