# Supplementary material for: Serum 5-Methyltetrahydrofolate Status Is Associated with One-Carbon Metabolism-Related Metabolite Concentrations and Enzyme Activity Indicators in Young Women
Source: Int J Mol Sci. 2023 Jul 1;24(13):10993. doi: 10.3390/ijms241310993 (PMC10341762; doi:10.3390/ijms241310993)
Supplement: Supplementary file 1 [file ijms-24-10993-s001.zip › SUP-2.pdf]

			5-MTHF	FA	Choline	Betaine	DMG	Methionine	SAM	SAH	tHcy	Cystathionine	tCys	Taurine	Serine	Glycine	Riboflavin
Low 5-MTHF	Folate cycle	5-MTHF	-														
group		FA	0.044	-													
(*n* = 113)	Choline metabolic	Choline	0.171	-0.039	-												
		Betaine	0.180	-0.042	0.199*	-											
		DMG	-0.016	-0.049	0.116	0.262**	-										
	Methionine cycle	Methionine	-0.115	-0.023	0.251**	0.110	0.141	-									
		SAM	0.109	0.160	0.231*	0.128	0.122	0.186*	-								
		SAH	-0.059	-0.178	0.484**	-0.070	0.174	0.218*	0.166	-							
		tHcy	-0.355**	-0.064	0.004	-0.401**	0.058	-0.025	0.030	0.168	-						
	Transsulfuration pathway	Cystathionine	-0.238*	-0.032	0.069	-0.034	0.167	0.378**	0.272**	0.139	-0.052	-					
		tCys	0.097	-0.021	0.090	-0.069	-0.042	0.009	0.223*	0.183	0.519**	-0.204*	-				
		Taurine	0.022	0.036	0.426**	-0.023	0.043	0.145	0.195*	0.477**	0.131	0.015	0.135	-			
	Amino acids	Serine	0.091	-0.117	0.402**	0.003	0.113	0.400**	0.073	0.266**	0.152	-0.042	0.185	0.295**	-		
		Glycine	-0.148	-0.095	0.131	-0.046	0.089	0.531**	-0.027	0.265**	0.125	-0.006	0.092	0.192*	0.408**	-	
	Vitamin	Riboflavin	-0.032	0.069	0.138	-0.023	-0.140	0.043	0.075	0.165	0.032	-0.120	0.068	0.089	0.057	0.211*	-
High 5-MTHF	Folate cycle	5-MTHF	-														
group		FA	0.073	-													
(*n* = 114)	Choline metabolic	Choline	0.026	-0.037	-												
		Betaine	0.156	0.007	0.403**	-											
		DMG	-0.102	-0.015	0.282**	0.296**	-										
	Methionine cycle	Methionine	0.049	-0.128	0.398**	0.286**	0.117	-									
		SAM	0.023	-0.118	0.182	0.082	0.100	0.071	-								
		SAH	-0.026	-0.018	0.325**	0.280**	0.147	0.219*	-0.030	-							
		tHcy	-0.377**	-0.019	0.170	-0.097	0.249**	0.031	-0.014	0.164	-						
	Transsulfuration pathway	Cystathionine	-0.222*	0.027	0.287**	0.266**	0.262**	0.371**	0.192*	0.271**	0.115	-					
		tCys	-0.076	-0.134	0.115	0.003	0.232*	0.097	0.039	0.229*	0.600**	0.097	-				
		Taurine	-0.026	-0.140	0.394**	0.156	0.113	0.175	0.124	0.277**	0.125	0.102	0.055	-			
	Amino acids	Serine	-0.166	-0.288**	0.197*	0.157	0.164	0.372**	-0.178	0.128	0.285**	0.264**	0.291**	0.195*	-		
		Glycine	0.023	-0.033	0.149	0.136	0.023	0.496**	-0.269**	-0.040	0.119	-0.062	0.102	0.054	0.393**	-	
	Vitamin	Riboflavin	0.119	0.122	0.060	0.080	-0.064	-0.103	0.112	0.067	-0.082	-0.098	0.014	0.058	-0.152	-0.050	-

Table S1. Correlation matrices between serum OCM-related metabolite concentrations stratified by dichotomous 5-MTHF concentrations.

Spearman correlation coefficient, asterisks indicate statistical significance of correlation coefficients as follows **$p < 0.01$; *$p < 0.05$.

Abbreviations, 5-MTHF; 5-methyltetrahydrofolate; FA; folic acid; DMG; dimethylglycine; SAM; *S* -adenosylmethionine; SAH; *S* -adenosylhomocysteine; tHcy; total homocysteine; tCys; total cysteine.

Serum homocysteic acid, pyridoxamine, and pyridoxine concentrations were below the limit of quantitation in all samples, so results are not shown.
